# The Design and Research of a New Hybrid Surface Plasmonic Waveguide Nanolaser

**DOI:** 10.3390/ma14092230

**Published:** 2021-04-26

**Authors:** Yahui Liu, Fang Li, Cheng Xu, Zhichong He, Jie Gao, Yunpeng Zhou, Litu Xu

**Affiliations:** School of Optical Information and Energy Engineering, Hubei Key Laboratory of Optical Information and Pattern Recognition and School of Mechanical and Electrical Engineering, Wuhan Institute of Technology, Wuhan 430073, China; 13212799763@163.com (Y.L.); xc578063977@163.com (C.X.); hzc_900503987@163.com (Z.H.); 21802010024@stu.wit.edu.cn (J.G.); z1433845556@163.com (Y.Z.); 15871430579@163.com (L.X.)

**Keywords:** hybrid plasmonic waveguide, finite element method, nanolaser

## Abstract

Using the hybrid plasmonic waveguide (HPW) principle as a basis, a new planar symmetric Ag-dielectric-SiO_2_ hybrid waveguide structure is designed and applied to nanolasers. First, the effects on the electric field distribution and the characteristic parameters of the waveguide structure of changes in the material, the nanometer radius, and the dielectric layer thickness were studied in detail using the finite element method with COMSOL Multiphysics software. The effects of two different dielectric materials on the HPW were studied. It was found that the waveguide performance could be improved effectively and the mode propagation loss was reduced when graphene was used as the dielectric, with the minimum effective propagation loss reaching 0.025. Second, the gain threshold and the quality factor of a nanolaser based on the proposed hybrid waveguide structure were analyzed. The results showed that the nanolaser has a lasing threshold of 1.76 μm^−1^ and a quality factor of 109 when using the graphene dielectric. A low-loss, low-threshold laser was realized, and the mode field was constrained by deep sub-wavelength light confinement. This structure has broad future application prospects in the integrated optics field and provides ideas for the development of subminiature photonic devices and high-density integrated circuits.

## 1. Introduction

Since the invention of the laser in the 1960s, lasers and other human inventions have had a major impact on all aspects of everyday life [1,2,3,4,5,6,7]. Recently, extensive research has been carried out on nanolasers based on nanotechnology. Traditional lasers use optical feedback resonators, which make it difficult for these lasers to be smaller in size than half their wavelength because of the diffraction limit [8]. The focusing and local field enhancement effects of surface plasmons [9] can enable realization of the functionality that light waves in free space cannot realize. Therefore, the surface plasmon nanolaser [10,11,12,13,14,15] can be used as a coherent nanoscale light source that is not bound or controlled by the diffraction limit. The size of this nanolaser is smaller than half its wavelength, which is the core aspect required to realize nanoscale optoelectronic integration [16,17,18,19,20]. However, these surface plasmonic waveguides suffer high losses during the transmission process because their structures contain metal materials that have a negative dielectric constant, which results in relatively short optical transmission distances [21,22,23] and hinders practical application of the devices. 

The emergence of the hybrid plasmonic waveguide (HPW) [24,25,26,27] presents a perfect solution to the contradiction described above. A low refractive index dielectric layer is inserted between the metal structure and the high refractive index dielectric structure. Then, the surface plasmon mode and the dielectric waveguide mode are coupled in the low refractive index gap, which causes the gap layer to play an energy storage role; thus, this type of hybrid waveguide has both a strong light field limiting ability and a low propagation loss. In 2009, the Xiang Zhang group at the University of California, Berkeley reported a deep-subwavelength hybrid plasmonic nanolaser based on nanowire structures [28]. This laser can confine light modes to a size that is 100 times smaller than the diffraction limit. In 2011, Bian et al. successfully designed a triangular wedge hybrid plasmonic waveguide structure based on previous research results [29]. Their wedge hybrid plasmonic waveguide mode is extremely small and is much smaller than the corresponding flat plate mode. In 2014, Zhang et al. reported a surface plasmon laser with a lasing wavelength of 370 nm at room temperature that was pumped using a laser with an operating wavelength of 355 nm, a pulse width of 10 ns, and a repetition rate of 100 kHz [30]. In 2020, Wang et al. presented a quantitative study of loss and gain in a plasmonic nanolaser [31]. Their hybrid plasmonic nanolaser performs well in terms of both size and luminescence intensity. However, the laser is currently at the exploration and development stage, and many problems have still to be solved in terms of its mechanism of action and heat dissipation [32,33,34]. Sun et al. [35] proposed a three-dimensional optical field limitation that greatly enhanced the optical field limitation of a plasmonic nanolaser when compared with that obtained using a two-dimensional plane. However, this work provided theoretical and simulation-based studies only. Some researchers have tried to use silicon/silicon oxide structures as the substrate for the hybrid waveguide structure and have applied this substrate together with mature complementary metal-oxide-semiconductor (CMOS) preparation technology [36,37], thus providing effective guidance that will help other researchers to prepare the waveguide structure. However, the area of the optical mode field is still similar in size to that of a conventional HPW. Therefore, simplification of the model structure, reduction of the manufacturing difficulty, and improvement of the localization ability of the optical field of the hybrid waveguide represent the key steps toward the miniaturization of surface plasmon devices.

In this paper, using COMSOL Multiphysics software, a new hybrid plasmonic waveguide model of the Ag-dielectric-SiO_2_ substrate structure is simulated using the finite element method. MgF_2_ and graphene are selected as the possible dielectric materials. The results show that the effective propagation loss of the hybrid plasmonic waveguide is smaller when graphene is used as the dielectric layer. A laser based on this structure can overcome the high losses of current structures, break the diffraction limit, and obtain a high-quality factor, which will then provide a valuable platform for further laser integration and miniaturization.

## 2. Characteristics of Symmetrical Ag-Dielectric-SiO_2_ Hybrid Waveguide

### 2.1. Theoretical Model of Waveguide Properties

The normalized mode area (SF), the effective propagation loss ($\alpha_{\mathrm{eff}}$), the effective refractive index ($n_{\mathrm{eff}}$), and the confinement factor (Γ) [38,39,40,41] are the most important basis parameters of the HPW nanolaser. Here, the effective refractive index is the real part of the relative propagation constant of the plasmonic waveguide, and the specific value can be calculated directly during the COMSOL Multiphysics simulation process, where the calculation formula is:

(1) $$n_{\mathrm{eff}}=\frac{\beta }{k_{0}},\;\beta =k_{0}\frac{\varepsilon_{1}^{\prime \prime }}{2{\left(\varepsilon_{1}^{\prime }\right)}^{2}}{\left(\frac{\varepsilon_{1}^{\prime }\varepsilon_{2}}{\varepsilon_{1}^{\prime }+\varepsilon_{2}}\right)}^{3/2}$$

where β is the propagation constant of the waveguide mode, $k_{0}$ is the wavevector in a vacuum, and $\varepsilon_{1}^{\prime \prime }$ and $\varepsilon_{1}^{\prime }$ are the real and imaginary parts of the metal’s dielectric constant, respectively. $\varepsilon_{2}$ is the dielectric constant of dielectric materials.

The effective mode field area parameter represents the capacity of the waveguide structure to limit the light field. Generally, a smaller mode field area indicates that the waveguide has a stronger light field limiting capacity. The ratio of the effective mode area $A_{\mathrm{eff}}$ to the diffraction limit area $A_{0}$ is defined as the normalized mode area. There is always a contradictory relationship between the normalized mode area and the transmission distance, and the optical field limiting capacity of a surface plasmonic waveguide can be represented more intuitively as shown in the following expression:

(2) $$SF=\frac{A_{\mathrm{eff}}}{A_{0}}=\frac{{\left(\iint {\left|E\right|}^{2}dxdy\right)}^{2}/\left(\iint {\left|E\right|}^{4}dxdy\right)}{\lambda^{2}/4}$$

where E is the electric field strength of the plasmon and λ is the wavelength of the light transmitted in the waveguide.

The energy confinement factor Γ represents the ratio of the electric field energy ($W_{s}$) in the semiconductor nanowires to the electric field energy (*W*) of the entire pattern. This factor is used to characterize the field strength-limiting capacity of the gain medium nanowires and can be expressed as follows:

(3) $$\Gamma =\frac{W_{s}}{W}=\frac{\iint_{s}W\left(r\right)dA}{{∭}_{\mathrm{all}}W\left(r\right)dA}.$$

### 2.2. Physical Model

**Figure 1:**
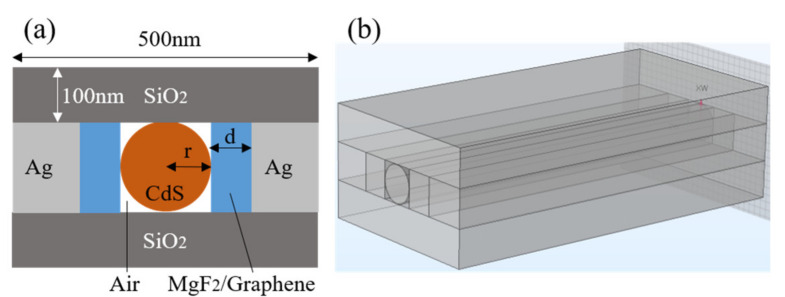
(**a**) The two-dimensional profile and (**b**) the three-dimensional geometry of waveguide model. The structure is composed of CdS gain medium nanowires, an upper SiO_2_ layer, a lower SiO_2_ layer, left and right medium layers and Ag layers, and an air gap area. Here, the dielectric layer thickness is *d* and the radius of the CdS nanowires is *r*. Based on the actual value synthesized in the laboratory and the difficulty of the simulation, the laser length *L* is set at 10 μm. The dielectric constants of CdS, MgF_2_, and SiO_2_ are 5.76, 1.96, and 2.18, respectively. The dielectric constants of graphene and silver were obtained via Drude model analyses and the calculated values are 2.2 + 4.8i and −9.2 + 0.3i, respectively.

The surface plasmon (SP) waveguide generated at the metal–dielectric surface is coupled with the cylindrical high refractive index gain dielectric waveguide to form a hybrid mode. When combined with the principle of the traditional silicon-on-insulator (SOI) waveguide with its excellent optical properties, the structural model of the new hybrid plasmonic waveguide designed in this work is as shown in Figure 1a. The structure is composed of CdS gain medium nanowires, an upper SiO_2_ layer, a lower SiO_2_ layer, left and right medium layers and Ag layers, and an air gap area. Here, the dielectric layer thickness is *d*, and the radius of the CdS nanowires is *r*. Based on the actual value of the waveguide synthesized by the laboratory and the difficulty of the simulation, the length *L* is set at 10 μm. In this design, we first analyzed the structural characteristics of MgF_2_, which is a traditional material, and then introduced the new dielectric material (graphene) to compare the pattern characteristics obtained when using the two different materials.

### 2.3. Modeling and Simulation

In this paper, COMSOL5.3 software is used by selecting the mode analysis function in the electromagnetic wave and frequency domain module to perform the required modeling, simulation, and numerical analysis. We decided to calculate the mixed mode of the hybrid waveguide using the eigenvalue solver after a comprehensive consideration of various factors. The real part of the eigenvalue represents the effective refractive index, and the imaginary part represents the propagation distance. The working wavelength of the hybrid structure is 490 nm. The dielectric constants of the CdS, MgF_2_, and SiO_2_ materials are 5.76, 1.96, and 2.18, respectively. The dielectric constants of graphene and silver were obtained via Drude model analyses [42,43], and the calculated values are 2.2 + 4.8i and −9.2 + 0.3i, respectively. The finite element method, which is widely used and highly respected by researchers, was used to construct the numerical model. We selected continuous boundary conditions inside the model and scattering boundary conditions outside the model. The boundary conditions were set as follows: the two end faces of the nanowire are the two mirrors of the resonator cavity, and the electromagnetic wave defined by the electric field is then added to either of these faces, while the other face is set as having no incident wave. To improve the mesh quality and ensure accurate calculations, the mesh at the interface between the dielectric layer and the nanowire is refined. Then, the mode characteristics and the gain thresholds in the modeled structure are simulated. The parameters and boundary conditions listed above are used in all model structures, and waveguide structures used to perform the nanolaser simulations in this paper.

## 3. Electric Field Distribution and Characteristic Parameters of Ag-MgF_2_-SiO_2_ Hybrid Waveguide

### 3.1. Electric Field Distribution

**Figure 2:**
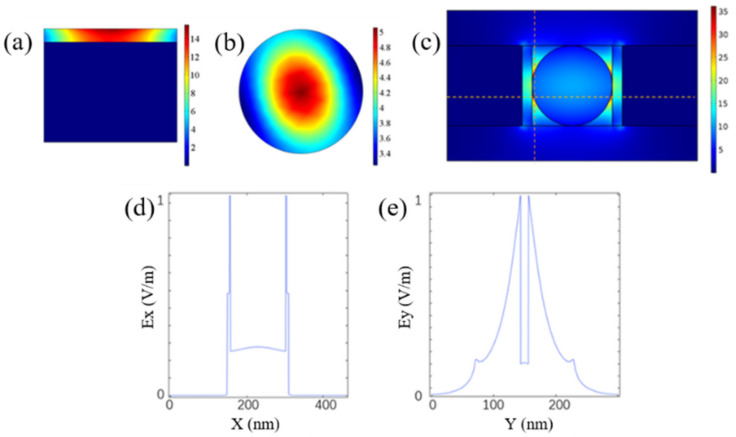
Electric field distributions of (**a**) SP mode, (**b**) nanowire waveguide mode, and (**c**) HPW mode. Normalized field intensities of (**d**) horizontal transverse and (**e**) longitudinal transverse modes.

To verify that the designed hybrid waveguide has both stronger field intensity and a stronger mode-limiting capability than the traditional SP waveguide, we simulated both the SP and nanowire waveguide modes and compared them with those of the HPW. As shown in Figure 2a–c, the electric field intensity of the hybrid waveguide is higher than the sum of the SP mode and the nanowire waveguide mode, thus indicating that the HPW structure can achieve strong coupling and that the mode area is limited to within a smaller range. Figure 2d,e shows the curves of the normalized electric field intensities of the horizontal transverse and longitudinal transverse modes in Figure 2c versus the changes in the transverse coordinates. The electric field energy is mainly concentrated at the junction between the dielectric layer and the nanowires and at the air gap.

**Figure 3:**
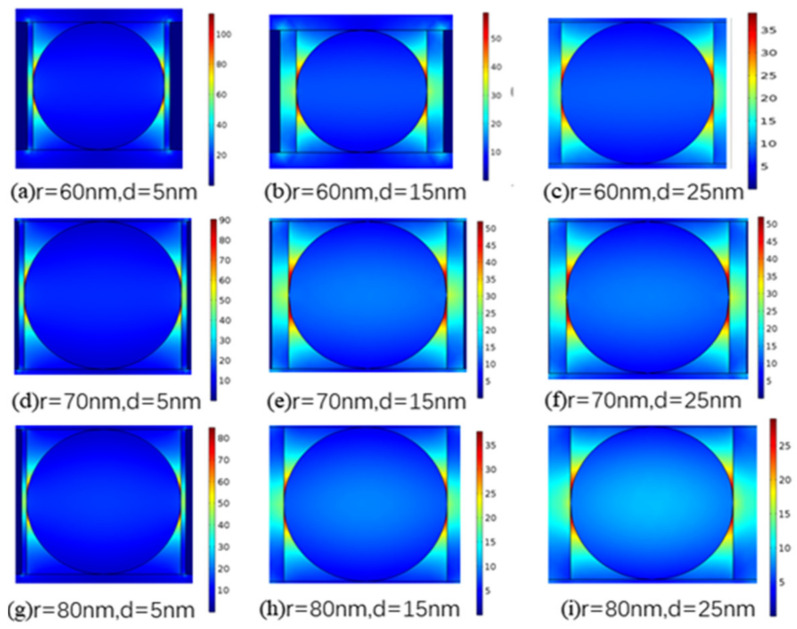
Electric field distributions for different values of nanowire radius *r* and dielectric layer thickness *d*. (**a**) r = 60 nm, d = 5 nm; (**b**) r = 60 nm, d = 15 nm; (**c**) r = 60 nm, d = 25 nm; (**d**) r = 70 nm, d = 5 nm; (**e**) r = 70 nm, d = 15 nm; (**f**) r = 70 nm, d = 25 nm; (**g**) r = 80 nm, d = 5 nm; (**h**) r = 80 nm, d = 15 nm; (**i**) r = 80 nm, d = 25 nm. When *r* is 60 nm and *d* is 5 nm, the electric field intensity has its highest value. When *d* and *r* increase, the electric field intensity decreases gradually. An increase in *r* causes the energy to converge gradually toward the nanowires.

Next, we analyzed the changes in the electric field distribution of the hybrid waveguide with variations in the geometric structure. Under the condition that the CdS nanowires had radii of 60, 70, and 80 nm, the mode field distributions of magnesium fluoride dielectric layers with thicknesses of 5, 15, and 25 nm were studied. As shown in Figure 3, when the nanowire radius *r* is 60 nm and the MgF_2_ thickness *d* is 5 nm, the electric field intensity reaches its highest value. With increases in *d* and *r*, the electric field intensity decreases gradually because thickening of the dielectric layer weakens the coupling effect between the SP mode and the nanowire mode. In terms of energy conservation, the electric field energy is dispersed into the medium layer, and the mode area gradually increases. An increase in *r* causes the energy to converge gradually toward the nanowires. It can also be seen from Figure 3a–i that an increase in the nanowire radius has little influence on the electric field, although an increase in the dielectric layer thickness will reduce the electric field intensity of the HPW significantly.

### 3.2. Discussion of Model Characteristic Parameters

**Figure 4:**
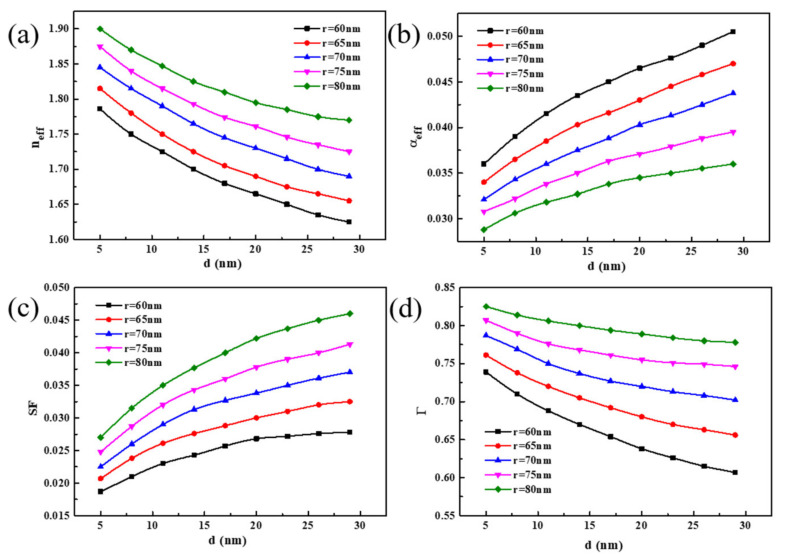
The mode properties change with the nanowire radius *r* and dielectric layer thickness *d*. (**a**) The effective index $n_{\mathrm{eff}}$, (**b**) the effective propagation loss $\alpha_{\mathrm{eff}}$, (**c**) the normalized model area SF and (**d**) the confinement factor Γ.

In this section, by varying the nanowire radius *r* and the MgF_2_ dielectric layer thickness d, the degree of influence of the geometric structure on the mode characteristic parameters was analyzed. The value of *r* was set to vary from 60 to 80 nm, while the value of *d* was set to range from 5 to 30 nm. As shown in Figure 4a, the effective refractive index $n_{\mathrm{eff}}$ of the HPW decreases gradually with increasing medium layer thickness. At a constant *d*, $n_{\mathrm{eff}}$ increases with increasing nanowire radius *r*. According to the results shown in Figure 4b, the effective propagation loss $\alpha_{\mathrm{eff}}$ increases with increasing *d*. However, when the *r* increases, $\alpha_{\mathrm{eff}}$ decreases gradually and reaches a minimum value of 0.029 when *d* is 5 nm and *r* is 80 nm. The results also show that the effective propagation loss is affected greatly by the size of the nanowire radius *r* relative to the dielectric layer thickness. Figure 4c shows that when the nanowire radius *r* and medium layer thickness *d* increase, the normalized model area SF also increases gradually. This is because the thickening of the dielectric layer weakens the coupling between the SP mode and the nanowire mode, which increases the SP mode losses in the metal and reduces the limiting effect of the dielectric layer on the mode field energy. An increase in *r* makes the nanowire waveguide mode stronger, and the energy is concentrated toward the nanowire, which leads to a gradual increase in the mode area. From Figure 4d, the value of the confinement factor *Γ* decreases with increasing *d* but increases in tandem with the nanowire radius, while its values within the selected geometrical size range were all greater than 60%; this demonstrates that most of the energy is contained in the CdS nanowires.

## 4. Electric Field Distribution and Characteristic Parameters of Ag-Graphene-SiO_2_ Hybrid Waveguide

### 4.1. Electric Field Distribution

**Figure 5:**
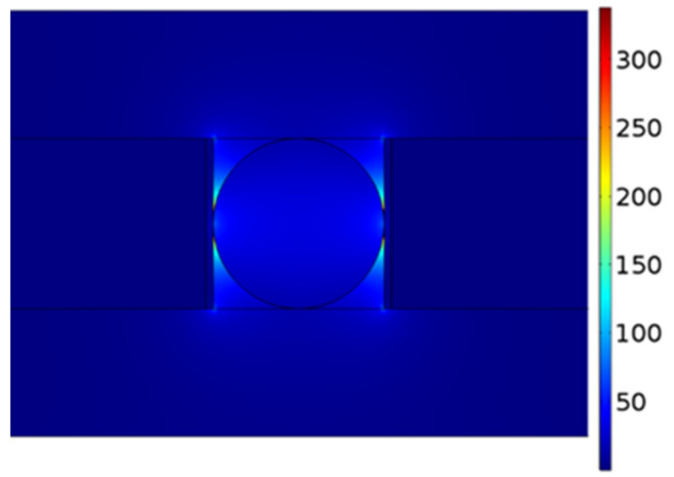
Waveguide electric field distribution with graphene as the dielectric layer.

In this section, we apply graphene, a relatively new material with excellent photoelectric properties, to the dielectric layer of the HPW, analyze the electric field distribution and mode characteristics of the structure, and compare it with the corresponding waveguide based on the MgF_2_ material layer. Since it is difficult to fabricate and simulate single-layer graphene in experiments and simulations, respectively, multilayer graphene is selected in this paper. To ensure that the optical properties of graphene are not reduced, the number of layers should not be too high. Therefore, we selected 5-nm-thick multilayer graphene as the dielectric material. As shown in Figure 5, when *r* is 80 nm, the electric field intensity of the waveguide mode is much higher than the corresponding maximum value for the device with the MgF_2_ dielectric layer shown in Figure 2.

### 4.2. Discussion of Model Characteristic Parameters

**Figure 6:**
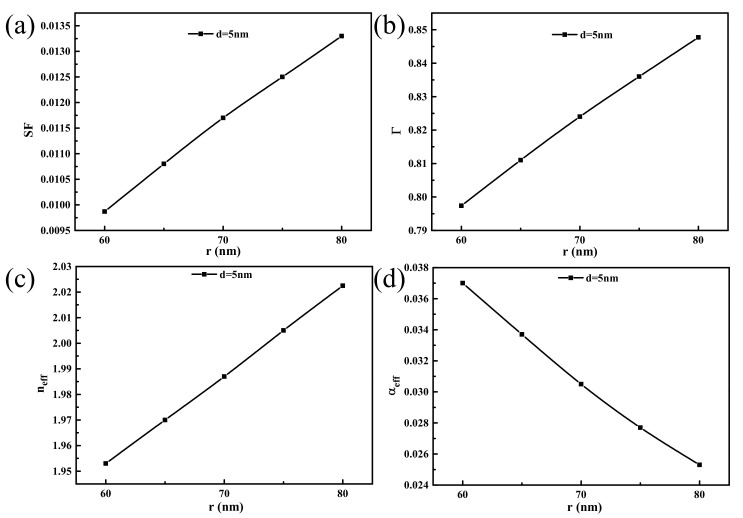
When dielectric layer thickness *d* = 5 nm, the mode properties change with the nanowire radius *r*. (**a**) The normalized model area SF, (**b**) the confinement factor Γ, (**c**) the effective index $n_{\mathrm{eff}}$ and (**d**) the effective propagation loss $\alpha_{\mathrm{eff}}$.

As shown in Figure 6, the graphene dielectric layer thickness was fixed at 5 nm and the waveguide mode characteristic parameters were then analyzed when the nanowire radius changed. The variation trends of the characteristic values of each model with the nanowire radius *r* are consistent with the analysis presented above. When compared with the results in Figure 4, the values of the effective refractive index $n_{\mathrm{eff}}$ and the confinement factor *Γ* increased obviously. The normalized mode area SF reaches a minimum of 0.0098 when the radius is 60 nm and the effective propagation loss $\alpha_{\mathrm{eff}}$ reaches a minimum of 0.025 when the radius is 80 nm. This is because the metal loss and the coupling loss, which are represented by the effective propagation loss and the modulus field area, respectively are a contradictory pair of physical quantities. To obtain better pattern characteristics, many researchers have balanced the contradictory relationship between these two quantities. Figure 6b,c show that when compared with the area of the normalized model, the nanowire radius *r* has a greater influence on the propagation loss, and the change trends of the two quantities are in opposite directions. Comprehensive analysis shows that the overall mode performance is optimal when *r* is 80 nm. This indicates that the graphene layer can improve the waveguide performance effectively and achieve the minimum coupling loss for the HPW.

## 5. Symmetrical Ag-Graphene-SiO_2_ Nanostructured Laser Based on the Characteristic Analysis

Using the graphene-dielectric hybrid plasmonic waveguide structure designed above, a 3D model was established in COMSOL Multiphysics, the electromagnetic wave frequency domain module was selected, and a boundary mode analysis was added for research. Scattering boundary conditions were added to the two end faces of the model, and one of these end faces was set as the incident end to simulate the nanolaser. The device designed in this paper will be pumped using a titanium sapphire laser (central wavelength of 405 nm and pulse width of 100 fs) and an emission wavelength of 490 nm can be realized at room temperature [44].

**Figure 7:**
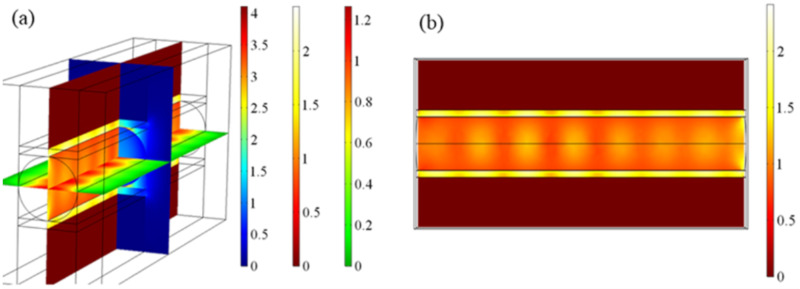
Three-dimensional simulation of hybrid waveguide nanolaser based on symmetric Ag-graphene-SiO2 substrate. (**a**) Three-dimensional simulation results; (**b**) Electric field distributions of yz cut planes.

Figure 7 shows the electric field distributions on the *xy*, *zx*, and *yz* sections of the Ag-graphene-SiO_2_ substrate-based hybrid waveguide nanolasers. Under excitation by the pump beam, SP waves are generated at the metal surface and pass through the graphene and the air gap before being coupled into the CdS nanowires, and CdS nanowires and the gain medium form a Fabry–Perot resonator; at this time, the gain medium is motivated to break the norm and the semiconductor materials combine with the electrons and valence band holes in the compound to enable the formation of stimulated radiation and realize population inversion and the optical amplification effect. Therefore, some of the SP waveguides and high-gain dielectric waveguides are coupled and stimulated from both ends of the nanowires after oscillation and amplification in the cavity. There are some light spots in the transverse fundamental mode, some bright spots in the wave hinterland, and corresponding dark spots in the node. The metal is completely dark, thus indicating that there is little energy distribution inside the metal, and the SP mode is separated from the high-dissipation metal layer.

To enable better evaluation of the performance of these nanolasers, we have studied the quality factor and the gain threshold from the main performance parameters. Among these parameters, the quality factor *Q* is an important parameter for evaluation of the performance of the resonant cavity and reflects the ability of the resonant cavity to bind photons. A larger *Q* value denotes a longer photon storage time, thus indicating that the attenuation of the resonant cavity is lower. The expression for *Q* is:

(4) $$Q=2\pi f\tau_{R}=2\pi f\frac{L}{\delta c}.$$

In Equation (4), f is the frequency of the optical field in the cavity, $\tau_{R}$ is the time constant of the cavity, *L* is the cavity length, *δ* is the cavity loss, and *c* is the speed of light in a vacuum [45]. This article analyzes the mirror loss in the resonant cavity only and does not study other loss types.

The gain threshold $g_{\mathrm{th}}$ is the minimum gain required to allow the laser to achieve stimulated emission and is the main reference index used to evaluate and measure the laser’s performance. Generally, a smaller threshold means that lower gain is required to achieve lasing and also indicates higher quality of laser operation. The expression for the gain threshold $g_{\mathrm{th}}$ is:

(5) $$g_{\mathrm{th}}=\left[k_{0}\alpha_{\mathrm{eff}}+\ln\left(1/R\right)/L\right]/\Gamma \left(n_{\mathrm{eff}}/n_{\mathrm{wire}}\right).$$

In Equation (5), *k*_0_ = 2π/*λ* is the wavenumber in a vacuum, $n_{\mathrm{wire}}$ is the refractive index of the CdS nanowires, $n_{\mathrm{eff}}$ is the real part of the effective refractive index of the mode, *Γ* is the confinement factor, and the scale factor $n_{\mathrm{eff}}/n_{\mathrm{wire}}$ represents the enhancement of the effective refractive index of the mode. In addition, ln(1/*R*)/*L* represents the mirror loss of the resonator. This equation considers the uniform non-absorption condition only and ignores the internal absorption and scattering losses of the nanowire [46,47,48,49]. Here, *R* is the end face reflectivity, which is defined as:

(6) $$R=\left(n_{\mathrm{eff}}-1\right)/\left(n_{\mathrm{eff}}+1\right).$$

**Figure 8:**
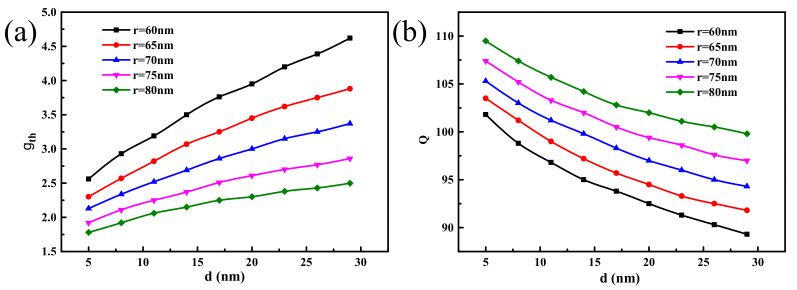
The change of (**a**) gain threshold $g_{\mathrm{th}}$ and (**b**) quality factor Q with the nanowire radius *r* and dielectric layer thickness *d*.

Figure 8 shows that the threshold $g_{\mathrm{th}}$ of the hybrid waveguide nanolaser with the symmetrical Ag–graphene–SiO_2_ substrate increases gradually as the thickness *d* of the graphene dielectric layer increases, but it also decreases gradually as the nanowire radius increases. The quality factor Q decreases gradually with increasing dielectric layer thickness *d* but gradually increases with increasing nanowire radius *r*. When *d* is 5 nm and *r* is 80 nm, $g_{\mathrm{th}}$ has a minimum value of 1.76 μm^−1^ and Q has a maximum value of 109. This is because any increase in the dielectric layer thickness reduces the coupling ability of the SP mode and the nanowire mode and also increases the SP mode loss in the metal, which then affects both the threshold and the quality factor.

Under the same conditions, the quality factor of the waveguide structure designed in this work is larger than that of the previous structure [50], and the comprehensive performance is better. When the restriction factor is increased by 20%, the propagation loss is reduced by 17%, and the area of the normalized mode is reduced by 21%. Our nanolaser has a larger quality factor, lower gain threshold, and better performance. Although our plasmon nanolaser has strong performance, there still are many shortcomings to be solved, mainly in the following aspects:

(1) The numerical aperture is small and the far field divergence angle is large.
(2) Lower power output.
(3) Low luminous efficiency and difficult control of beam quality.

## 6. Conclusions and Development

A plane symmetrical Ag–dielectric–SiO_2_ hybrid waveguide structure is designed. Using COMSOL Multiphysics software, the finite element method is used to study the normalized mode scaling factor (SF), the effective refractive index $n_{\mathrm{eff}}$, the confinement factor Γ, and the effects of different dielectric materials on the performance of the designed waveguide. Then, the quality factor *Q* and the gain threshold $g_{\mathrm{th}}$ of the nanolaser designed on the basis of this structure are analyzed. The best waveguide performance of this structure is achieved when *r* = 80 nm. When the waveguide structure is used in a laser, the optical field distribution in the cavity is both stable and concentrated and the laser has a small gain threshold and a large quality factor. Following comprehensive consideration, we selected graphene with *d* = 5 nm as the dielectric layer along with nanowires with the optimal size of *r* = 80 nm. At this time, the effective propagation loss is as low as 0.025. When the dielectric material is graphene, the parameter values of *r* = 80 nm and *d* = 5 nm give the best performance for the laser based on this structure, with $g_{\mathrm{th}}$ = 1.76 μm^−1^ and *Q* = 109. This research is expected to aid in the miniaturization and integration of photonic equipment and is expected to have broad application prospects in fields including biology, medicine, and optical communications.

With the requirement of new and miniaturization of plasmon nanolasers [51,52], the development trend of nanolasers can be seen in the following aspects:

(1) The pumping could potentially be developed from optical excitation to electrical injection for easier operation.
(2) The resonator activates the medium from the simple structure of a single nanowire to the complex structure of nanowire array or quantum dots.
(3) In the aspect of existence form, it develops from independent monomer structure to integrated array form.

With the progress of nanomachining technology and characterization technology, we believe that it is necessary to further optimize the design of nanomachining plasma laser, optimize the processing technology, improve the key technology of testing the emission characteristic parameters of laser pump, and optimize the device performance. Plasma nanolasers provide reliable laser sources for applications in sensing [53,54], diagnostics, biomedicine, telecommunications, absorbers, measurements, nanoelectronics, automotive, and other related fields [55], and provide technical support for the integrated development of related information technology.

## Data Availability

Not applicable.
